# *CGB5* expression is independently associated with poor overall survival and recurrence‐free survival in patients with advanced gastric cancer

**DOI:** 10.1002/cam4.1364

**Published:** 2018-02-23

**Authors:** Yuxin Yang, Yonghong Shi, Yanjuan Hou, Ying Lu, Jinliang Yang

**Affiliations:** ^1^ Department of Gastroenterology Cangzhou Central Hospital Cangzhou 061001 China; ^2^ Department of Pathophysiology Hebei medical university Shijiazhuang 050000 China; ^3^ Cancer Center West China Medical School West China Hospital Sichuan University Chengdu 610041 China; ^4^ Guangdong Zhongsheng Pharmaceutical Co., Ltd. Guangdong 523325 China

**Keywords:** *CGB5*, gastric cancer, hCG*β*, overall survival, recurrence‐free survival

## Abstract

The human *CGB5* gene encodes chorionic gonadotropin (hCG)*β* 5, which is aberrantly expressed in trophoblastic neoplasm and in some non‐trophoblastic neoplasms. Fucntional studies observed that it involved tumor initiation, growth, and metastatic outgrowth. In this study, using data from the International Cancer Genome Consortium (ICGC) and the Cancer Genome Atlas (TCGA)‐stomach adenocarcinoma (STAD), we assessed the independent prognostic value of *CGB5* expression in patients with primary gastric cancer (GC). Results showed that *CGB5* expression was nearly not expressed in normal GC tissues. In comparison, its expression was detected in 214 of the 415 primary GC cases (51.6%) in TCGA‐STAD and was associated with poor response to primary therapy and a higher risk of recurrence and death. In early stages, *CGB5* expression was not a prognostic factor in terms of OS (HR: 1.448; 95% CI: 0.811–2.588, *P = *0.211) or RFS (HR: 1.659; 95% CI: 0.778–3.540, *P = *0.190). However, its expression was independently associated with unfavorable OS (HR: 1.719; 95% CI: 1.115–2.651, *P = *0.014) and RFS (HR: 3.602; 95% CI: 1.708–7.598, *P = *0.001) in advanced stages. Using deep sequencing data from TCGA‐STAD *,* we found that *CGB5* expression was not related to its genetic amplification or DNA methylation in GC. Based on these findings, we infer that *CGB5* expression is common in GC patients and its expression might independently predict poor OS and RFS in advanced stages, but not in early stages of GC.

## Introduction

Human chorionic gonadotropin (hCG) is a glycoprotein hormone that plays an important role during pregnancy, such as modulation of implantation, placentation, placental angiogenesis, and maternal/fetal immune responses 1. As a glycoprotein hormone, hCG is a heterodimers consisting of a common *α*‐subunit and an unique *β*‐subunit which confers biological specificity. Previous studies found that the upregulation of free hCG*β* is a marker of the trophoblastic neoplasm, such as choriocarcinoma 2 and its aberrant expression was also observed in some non‐trophoblastic neoplasms including endometrial carcinoma and ovarian 3, testicular 4, breast cancer 5, 6, and gastric carcinomas 7.

There are six genes clustered on chromosome 19q13.3 encoding the *β*‐subunit, including *CGB1, CGB2, CGB3, CGB5, CGB7,* and *CGB8. CGB1* and *CGB2* might encode a protein unrelated to hCG, while the rest four genes encode the two specific hCG*β* proteins. *CGB7* encodes a protein with an alanine at position 117, while *CGB3, CGB5,* and *CGB8* encode an aspartic acid at this position 8. According to this difference, *CGB7* was classified into type I gene, while the other three (*CGB3*,* CGB5,* and *CGB8*) were classified into type II genes 9. A series of previous studies found that dysregulated type II genes are involved in some tumor initiation, growth, and metastatic outgrowth 10, such as colorectal cancer 11 and ovarian cancer 12, 13. Among the type II genes, the oncogenic mechanisms of aberrantly expressed *CGB5* have been characterized in ovarian cancer 12, 13.

hCG*β* expression also has a prognostic value in some cancers. In urothelial carcinomas, hCG*β* can potentially be used as a marker of patients’ clinical response to treatment 14. Elevated serum hCG*β* and aberrant p53 expression were strongly associated with poor prognosis of serous ovarian carcinoma 3. One early study based on 54 patients with gastric cancer (GC) found that hCG*β*‐positive cells can be found in the gastric tumor by immunohistochemical (IHC) staining 15. However, the expression profile of *CGB5* and its prognostic value in GC remains obscure. In this study, using data from the Cancer Genome Atlas (TCGA), we assessed the independent prognostic value of the *CGB5* expression in patients with primary GC.

## Materials and Methods

### Data mining in the International Cancer Genome Consortium (ICGC) and the Cancer Genome Atlas (TCGA)

The ICGC was launched in 2008 to coordinate large‐scale cancer genome studies in tumors from 50 cancer types and/or subtypes 16. In the specimen‐centric database, 371 primary GC cases with intact OS data were recorded. The OS data were downloaded using the UCSC Xena browser (https://xenabrowser.net/). In TCGA‐Stomach Adenocarcinoma (STAD), 415 GC samples and 35 normal gastric samples were included. Among the 415 patients, 388 cases had intact OS data recorded. The level‐3 data, including *CGB5* expression (RNAseq ‐ IlluminaHiSeq UNC), age at initial diagnosis, gender, pathological stage, histological grade, radiation therapy, targeted molecular therapy, *Helicobacter pylori* infection, primary therapy outcome, residual tumor, recurrence status, and living status in this cohort, were also obtained using the UCSC Xena browser. Kaplan–Meier curves of OS and recurrence‐free survival (RFS) after primary therapy were generated by GraphPad Prism v6.0 (GraphPad Inc.).

*CGB5* DNA methylation (Illumina 450k infinium methylation beadchip) and gene‐level thresholded GISTIC2‐processed copy‐number data, which defines genetic changes as homozygous deletion (−2), heterozygous loss −1), copy‐neutral (0), low‐level copy gain (+1), high‐level amplification (+2) were also downloaded from the Xena browser.

### Examining of CGB5 protein expression

*CGB5* expression at the protein level in normal human tissues and in cancer tissues was examined using IHC staining data in the Human Protein Atlas (HPA) (http://www.proteinatlas.org/) 17, 18.

### Statistical analysis

Gastric cancer patients were divided into *CGB5* expression positive (>0) and negative (=0) groups. Statistical analysis was performed using GraphPad Prism v6.0 and SPSS 19.0 (SPSS Inc. Chicago, IL, USA). Continuous variables were reported as means ± standard deviation (SD). The group difference was compared by two‐tailed Student's *t*‐test or ANOVA with Student–Newman–Keuls test as a post hoc test. The association between *CGB5* expression and the clinicopathological characteristics was evaluated using *χ* ^2^ tests. Log‐rank test was performed to assess the significance of the difference between OS/RFS curves. The prognostic values of *CGB5* expression in terms of OS and RFS were analyzed by univariate and multivariate Cox regression models. Linear regression analysis was conducted to assess the correlation between *CGB5* expression and its DNA methylation. *P *<* *0.05 was considered statistically significant.

## Results

### *CGB5* expression profiles in GC and normal gastric tissues

**Figure 1:**
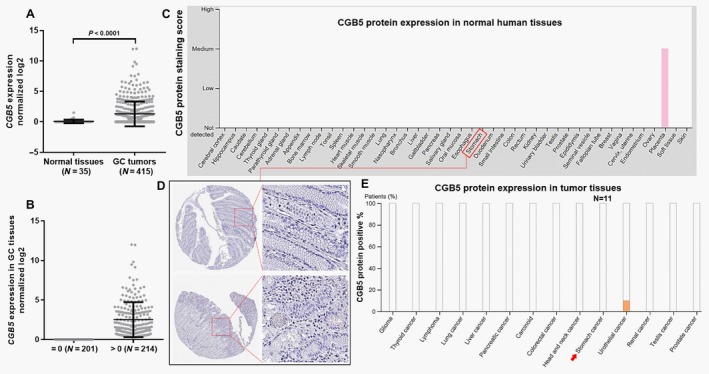
Comparison of *CGB5* expression in different patient groups. (A) Comparison of *CGB5* expression between GC cancer (*N* = 415) and normal gastric tissues (*N* = 35). (B) The expression profile of *CGB5* in 415 patients. (C) CGB5 protein expression summary in normal human tissues. Data were obtained from: http://www.proteinatlas.org/ENSG00000189052-CGB5/tissue. (D) Representative images of CGB5 IHC staining in normal gastric tissues. (E). CGB5 protein expression summary in some human cancer. Data were obtained from: http://www.proteinatlas.org/ENSG00000189052-CGB5/pathology.

By comparing *CGB5* expression in TCGA‐STAD, we found that *CGB5* expression was significantly higher in GC tissues (*N* = 415) than in normal gastric tissues (*N* = 35) (Fig. 1A). Among the 415 cases of GC, 214 cases (51.6%) had *CGB5* expression (Fig. 1B). By examining CGB5 protein expression in the HPA, we found that CGB5 protein was nearly not detectable in all normal human tissues, except in placenta (Fig. 1C). In normal gastric glandular cells, CGB5 was not detectable by IHC staining (Fig. 1D). In comparison, in 11 cases of GC tissues examined by CGB5 antibody (HPA038934), not positive staining was observed (Fig. 1E, red arrow). However, due to small number of cases examined, we could not exclude the possibility that some GC tumors might be CGB5 positive.

### Comparison of *CGB5* expression in different GC patient groups

**Figure 2:**
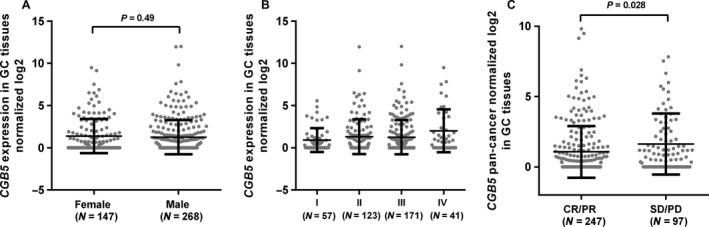
Comparison of *CGB5* expression in different GC patient groups. (A–C) Comparison of *CGB5* expression between female and male patients (A), in different pathological stages (B) and in patients with responses (CR+PR) and without responses (SD+PD) (C).

By comparing *CGB5* expression between patients with different clinicopathological parameters, we did not find significant difference between female and male patients (Fig. 2A) and among different stages of diseases (Fig. 2B). However, the patients with overall responses to primary therapy [complete remission (CR) and partial remission (PR)] had significantly lower *CGB5* expression (Fig. 2C).

Then, we compared the clinical characteristics between the *CGB5‐*positive (>0) and *CGB5‐*negative (=0) groups (Table 1). Results showed that the *CGB5*‐positive group had a lower overall response rate (CR and PR] (110/173, 63.6%) than the *CGB5*‐negative group (137/171, 80.1%) (*P = *0.0007; Table 1). In addition, we also observed significantly higher ratios of recurrence after primary therapy (49/161, 30.4%) and death (93/199, 46.7%) in the *CGB5*‐positive group compared with the negative group (23/163, 14.1%, and 64/189, 33.9%) (*P = *0.0004 and 0.0098, respectively; Table 1).

**Table 1:** The association between *CGB5* expression and the clinical parameters in patients with primary GC in TCGA‐STAD

Parameters	*CGB5* expression		*χ* ^2^	*P* value
	>0 (*N* = 214)	=0 (*N* = 201)		
Age (Mean ± SD)	65.43 ± 10.48	65.87 ± 10.92		0.68
Gender				
Female	79	68	0.43	0.51
Male	135	133		
Pathological stage				
I/II	92	88	0.023	0.88
III/IV	110	102		
Discrepancy+null	12	11		
Histological grade				
G1/G2	82	78	0.024	0.88
G3	128	118		
GX	4	5		
Radiation therapy				
No	153	147	0.15	0.70
Yes	38	33		
Discrepancy+null	23	21		
Targeted molecular therapy				
No	94	102	1.80	0.18
Yes	94	77		
Discrepancy+null	26	22		
*H. pylori* infection				
No	81	76	0.95	0.33
Yes	8	12		
Null	125	113		
Primary therapy outcome				
CR+PR	110	137	11.61	0.0007
SD+PD	63	34		
Discrepancy+null	41	30		
Residual tumor				
R0	166	164	0.90	0.34
R1 + R2	20	14		
RX+null	28	23		
Recurrence status				
No	112	140	12.49	0.0004
Yes	49	23		
Null	53	38		
Living status				
Living	106	125	6.67	0.0098
Dead	93	64		
Null	15	12		

GX, grade cannot be assessed; CR, complete remission; PR, partial remission; SD, stable disease; PD, progressive disease; R0, No residual tumor; R1, Microscopic residual tumor; R2, Macroscopic residual tumor; RX, The presence of residual tumor cannot be assessed; null, no data.

### *CGB5* expression was independently associated with poor OS in patients with advanced GC

**Figure 3:**
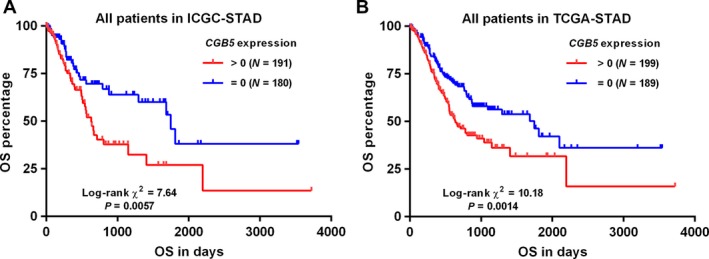
Kaplan–Meier curves of OS in GC patients. (A–B) Kaplan–Meier curves of OS in GC patients. Survival curves were generated using data from ICGC (A) and TCGA (B). Patients were divided into *CGB5‐*positive (>0) and negative (=0) groups.

**Figure 4:**
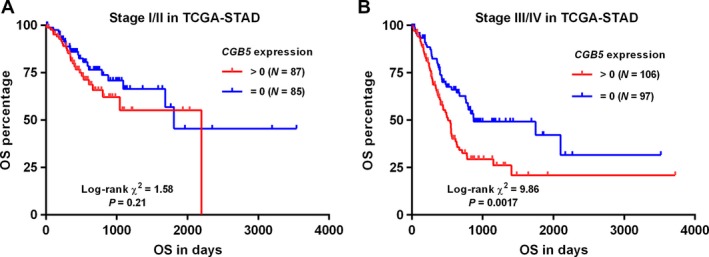
Kaplan–Meier curves of OS in early and advanced stages of GC patients. (A–B) Kaplan–Meier curves of OS in early stages group (I/II) (A) or in advanced stages group (III/IV) (B). Data were generated using data from TCGA‐STAD. Patients were divided into *CGB5‐*positive (>0) and negative (=0) groups.

To explore the association between CGB5 expression and OS in GC patients, we used both data from ICGC and TCGA. By generating Kaplan–Meier curves of OS, we found that *CGB5* expression (>0) was associated with shorter OS in primary GC patients, no matter in ICGC (*P = *0.0057) (Fig. 3A) or in TCGA‐STAD (*P = *0.0014) (Fig. 3B). However, in subgroup analysis, we only confirmed the association in advanced stages (stage III/IV) (*P = *0.0017) (Fig. 4B), but not in early stages (stage I/II) (*P = *0.21) (Fig. 4A). To further investigate the independent prognostic value of *CGB5* in terms of OS, univariate and multivariate analysis based on the COX regression model was conducted. In early stages, *CGB5* expression was not a prognostic factor (HR: 1.448; 95% CI: 0.811–2.588, *P = *0.211; Table 2). However, its expression was independently associated with poor OS in advanced stages (HR: 1.719; 95% CI: 1.115–2.651, *P = *0.014; Table 3).

**Table 2:** Univariate and multivariate analysis of OS/RFS in stage I/II patients in TCGA‐STAD

Parameters	Univariate analysis				Multivariate analysis			
	*P*	HR	95% CI (lower/upper)		*P*	HR	95% CI (lower/upper)	
OS								
Age (Continuous)	0.495	1.010	0.982	1.038				
Gender Female vs. Male	0.473	0.798	0.431	1.478				
Histological grade G3 vs. G1/G2	0.080	1.721	0.937	3.162				
Radiation therapy No vs. Yes	0.521	1.330	0.557	3.174				
Targeted molecular therapy No vs. Yes	0.761	0.911	0.498	1.665				
*H. pylori* infection No vs. Yes	0.562	1.829	0.238	14.081				
Primary therapy outcome SD/PD vs. CR/PR	0.000	3.333	1.726	6.437				
*CGB5* expression >0 vs. =0	0.211	1.448	0.811	2.588				
RFS								
Age (Continuous)	0.713	1.006	0.973	1.040				
Gender Female vs. Male	0.047	0.446	0.201	0.988	0.216	0.593	0.259	1.358
Histological grade G3 vs. G1/G2	0.174	1.631	0.806	3.299				
Radiation therapy No vs. Yes	0.516	1.420	0.493	4.089				
Targeted molecular therapy No vs. Yes	0.581	0.825	0.416	1.636				
Primary therapy outcome SD/PD vs. CR/PR	0.000	4.624	2.217	9.643	0.001	3.581	1.637	7.836
*CGB5* expression >0 vs. =0	0.032	2.197	1.070	4.512	0.190	1.659	0.778	3.540

G1, well differentiated (low grade); G2, moderately differentiated (intermediate grade); G3, poorly differentiated (high grade); CR, complete remission; PR, partial remission; SD, stable disease; PD, progressive disease.

**Table 3:** Univariate and multivariate analysis of OS/RFS in stage III/IV patients in TCGA‐STAD

Parameters	Univariate analysis				Multivariate analysis			
	*P*	HR	95% CI (lower/upper)		*P*	HR	95% CI (lower/upper)	
OS								
Age (Continuous)	0.001	1.035	1.015	1.056	0.089	1.019	0.997	1.042
Gender Female vs. Male	0.716	0.925	0.609	1.407				
Histological grade G3 vs. G1/G2	0.210	1.313	0.858	2.009				
Radiation therapy No vs. Yes	0.000	3.663	1.974	6.796	0.064	1.954	0.962	3.971
Targeted Molecular therapy No vs. Yes	0.000	2.240	1.472	3.408	0.051	1.637	0.999	2.682
*H. pylori* infection No vs. Yes	0.188	1.868	0.737	4.734				
Primary therapy outcome SD/PD vs. CR/PR	0.000	2.811	1.804	4.379	0.011	1.858	1.155	2.988
Residual tumor R1/R2 vs. R1	0.000	2.576	1.577	4.207	0.000	2.594	1.528	4.404
*CGB5* expression >0 vs. =0	0.002	1.918	1.281	2.870	0.014	1.719	1.115	2.651
RFS								
Age (Continuous)	0.376	0.988	0.961	1.015				
Gender Female vs. Male	0.128	0.543	0.247	1.193				
Histological grade G3 vs. G1/G2	0.088	1.999	0.903	4.426	0.049	2.362	1.003	5.565
Radiation therapy No vs. Yes	0.015	3.174	1.257	8.018	0.040	2.841	1.048	7.703
Targeted Molecular therapy No vs. Yes	0.467	0.767	0.374	1.570				
*H. pylori* infection No vs. Yes	0.586	1.522	0.336	6.900				
Primary therapy outcome SD/PD vs. CR/PR	0.000	3.686	1.812	7.500	0.006	2.810	1.338	5.901
Residual tumor R1/R2 vs. R0	0.283	1.688	0.650	4.386				
*CGB5* expression >0 vs. =0	0.000	3.758	1.830	7.716	0.001	3.602	1.708	7.598

G1, well differentiated (low grade); G2, moderately differentiated (intermediate grade); G3, poorly differentiated (high grade); CR, complete remission; PR, partial remission; SD, stable disease; PD, progressive disease; R0, no residual tumor; R1, microscopic residual tumor; R2, macroscopic residual tumor.

### *CGB5* expression was independently associated with poor RFS in patients with advanced GC

**Figure 5:**
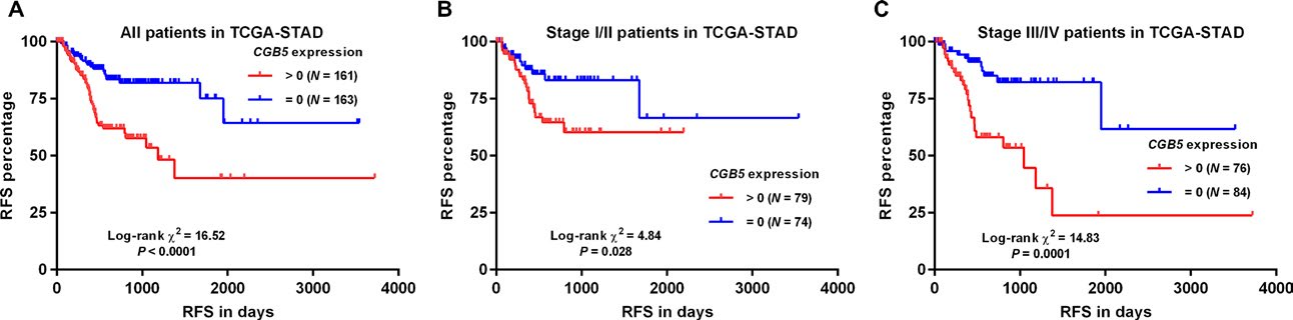
Kaplan–Meier curves of RFS in GC patients. (A–C) Kaplan–Meier curves of RFS in all patients (A), in early stages group (I/II) (B) and advanced stages group (III/IV) (C). Data were generated using data from TCGA‐STAD. Patients were divided into *CGB5‐*positive (>0) and negative (=0) groups.

Using RFS as an outcome indicator, we found that *CGB5* expression was associated with poor RFS (*P < *0.0001) (Fig. 5A). Subgroup analysis showed that the association was significant in both early (*P = *0.028) (Fig. 5B) and advanced stages (*P = *0.0001) (Fig. 5C). However, *CGB5* expression was not an independent prognostic factor of RFS in early stages (HR: 1.659; 95% CI: 0.778–3.540, *P = *0.190; Table 2). In comparison, its expression was independently associated with unfavorable RFS in advanced stages (HR: 3.602; 95% CI: 1.708–7.598, *P = *0.001; Table 3).

### *CGB5* expression was not modulated by genetic amplification or DNA methylation in GC

**Figure 6:**
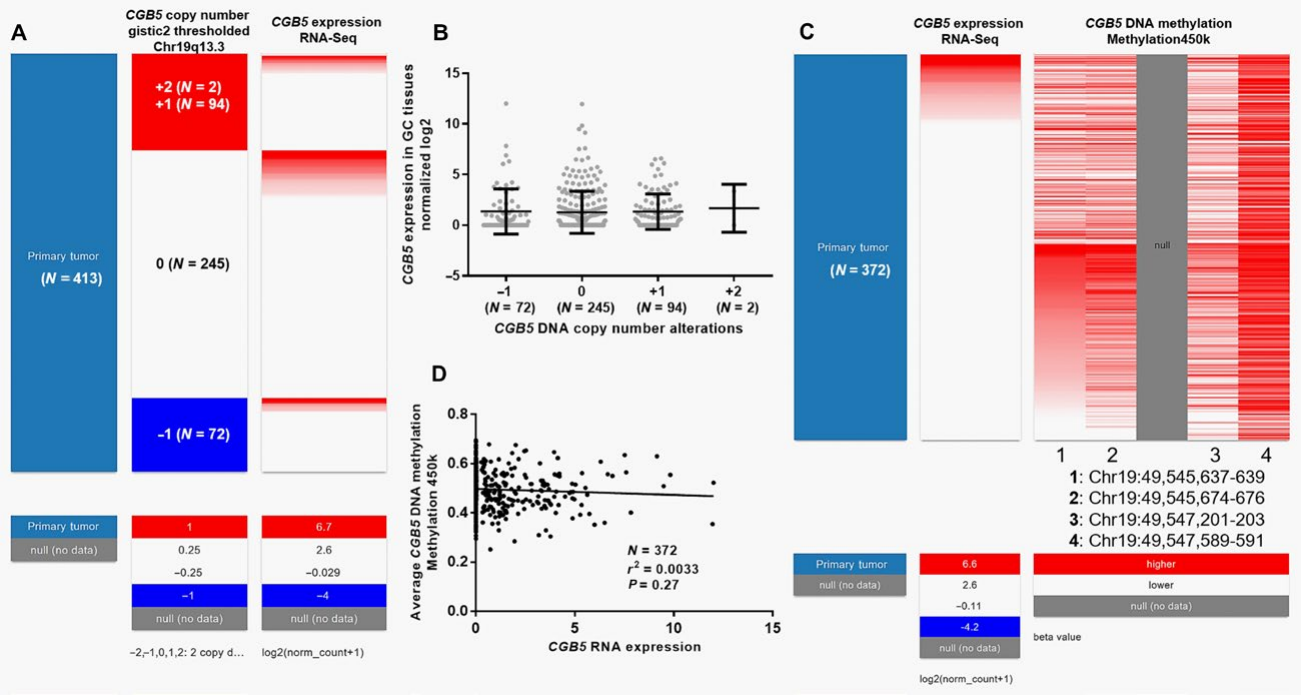
The association between *CGB5* expression and its DNA amplification and methylation. (A–B) Heatmap (A) and plots chart (B) of *CGB5* expression in groups with different genetic alterations. −1: heterozygous loss, 0: copy‐neutral, +1: low‐level copy gain, and +2: high‐level amplification. Heatmap (C) and regression analysis (D) of the correlation between *CGB5* DNA methylation and its RNA expression.

Then, we tried to explore the mechanisms of *CGB5* dysregulation using deep sequencing data from TCGA‐STAD. A total of 413 patients had DNA amplification and *CGB5* expression measured at the same time (Fig. 6A). No significant difference was observed in different DNA amplification groups (Fig. 6B). A total of 372 patients had *CGB5* DNA methylation and RNA expression measured simultaneously (Fig. 6C). Regression analysis showed that there was no significant correlation between *CGB5* DNA methylation and its RNA expression (*P = *0.27, Fig. 6D).

## Discussion

Ectopic expression of hCG*β* has been associated with malignant behaviors in non‐trophoblastic tumors 19. As *CGB5* is one of the key hCG*β* encoding genes, we examined its expression profile in GC. Interestingly, our data showed that its expression was nearly not expressed in normal GC tissues. In comparison, its expression was detected in 214 of the 415 primary GC cases (51.6%) in TCGA‐STAD, suggesting that *CGB5* expression was common among the patients. By examining CGB5 protein expression in the HPA, we found that CGB5 protein was not detectable in most of normal human tissues, including normal gastric tissues. Although CGB5 expression was not detected in 11 cases of GC tissues in the database, we could not exclude the possibility that some GC tumors might be CGB5 positive. Besides, we also found that its aberrant expression was significantly related to poor therapeutic responses. Therefore, in the future, it is meaningful to explore the possible therapeutic value of CGB5‐targeting drugs, such as anti‐CGB5 or antibody‐drug conjugate (ADC) 20, 21, in the potential CGB5‐positive cases.

Previous studies found that the structure of hCG*β* shows significant morphological similarity with that of the “cystine knot growth factor” (CKGF) family members such as transforming growth factor *β* (TGF*β*), platelet‐derived growth factor B (PDGFB), nerve growth factor (NGF), and vascular endothelial growth factors (VEGFs). The structural similarity suggests that there might be cross talk between these growth regulatory systems 22, 23. In fact, recent studies demonstrated that hCG acts as a proangiogenic factor in some tumors, which is similar to VEGF 22, 23. In ovarian cancer, *CGB5* could enhance vasculogenic mimicry formation and upregulate the expression of the vascular markers CD31 12, 24. In addition, its upregulation also suppresses the apoptosis of the cancer cells by decreasing *B‐cell lymphoma 2* (*BCL2*) and increasing *BCL2‐associated X protein* (*BAX*), and *baculoviral IAP repeat containing 5* (*BIRC5*) transcription 13. In addition, HCG*β* can also modulate the expression of epithelial‐to‐mesenchymal transition (EMT)‐related genes, including suppressing E‐cadherin and increasing phospho‐SMAD2, SNAIL and TWIST in colorectal cancer cells, the effects of which are similar to that of TGF*β* 11. These findings suggest that hCG*β* can induce EMT via the TGF*β* signaling pathway. These mechanisms might help to explain why hCG*β* upregulation is associated with malignant tumor behaviors.

Currently, clinicopathologic staging is the most important indicator of resectability and prognosis for GC. However, significant variations in response to primary therapies have been observed in patients with the same or similar stages 25, 26. Therefore, it is meaningful to explore other potential biomarkers of prognosis. Previous studies found that the serum hCG*β* level has prognostic values in some cancers. It is an independent prognostic factor in urothelial transitional cell carcinoma (TCC) patients receiving chemotherapy for urothelial TCC in both curative and palliative settings 27. The OS in hepatocellular carcinoma patients with low serum concentrations of hCG*β* is statistically and significantly better than in patients with elevated concentrations 28. Serum hCG*β* level has been shown to be associated with unfavorable prognosis in colorectal cancer 11. In this study, we also examined the prognostic value of *CGB5* in GC using data from two large databases (ICGC and TCGA). Our secondary analysis showed that that *CGB5* expression was associated with higher ratios of recurrence and death in GC patients. By performing univariate and multivariate analysis based on the COX regression model, we confirmed that *CGB5* expression was independently associated with inferior OS and RFS in advanced stages, but not in early stages of GC. Therefore, we infer that *CGB5* expression might serve as a valuable prognostic marker in advanced GC patients. DNA amplification or hypomethylation are two common mechanisms of upregulated oncogenes in GC 29, 30, 31, 32. Using deep sequencing data from TCGA‐STAD, we failed to identify any significant associations between *CGB5* expression and its DNA amplification or methylation. These results excluded the possibility of two common mechanisms of gene dysregulation in aberrant *CGB5* expression in GC. Therefore, the exact mechanism of *CGB5* expression should be explored in the future. In addition, although we showed the prognostic value of *CGB5* expression, more studies are required to characterize the mechanism underlying its expression and GC development and/or therapeutic responses. Elucidation of the *CGB5‐*related signaling pathways is beneficial for future exploration of targeted therapeutic strategies.

## Conclusion

*CGB5* expression is common in GC patients, and its expression might independently predict poor OS and RFS in advanced stages, but not in early stages of GC.

## Conflict of Interest

The authors have no conflict of interest.
